# Flavonoids and *Devosia* sp SL43 cell-free supernatant increase early plant growth under salt stress and optimal growth conditions

**DOI:** 10.3389/fpls.2022.1030985

**Published:** 2022-11-10

**Authors:** Ateeq Shah, Sowmyalakshmi Subramanian, Donald L. Smith

**Affiliations:** Department of Plant Sciences, McGill University, Montreal, QC, Canada

**Keywords:** salt stress, biostimulants, flavonoids, cell-free supernatant, plant abiotic stress, canola, soybean, plant growth

## Abstract

Salt stress is a major threat to modern agriculture, significantly affecting plant growth and yield, and causing substantial economic losses. At this crucial time of increasing climate change conditions, soil salinity will continue to develop and become an even more serious challenge to crop agriculture. Hence, there is a pressing need for sustainable techniques in agricultural production that could meet the dual challenges of crop productivity and environmental instability. The use of biostimulants in agricultural production has greatly influenced plant health and global food production. In particular, the application of bioactive materials produced by beneficial microbes is becoming a common practice in agriculture and provides numerous benefits to plant growth and resistance to stressful conditions. In this research two biostimulants; a type of plant secondary metabolite (flavonoids) and a microbe-based material (CFS: Cell-Free Supernatant) containing active compounds secreted by a novel bacterial strain isolated from *Amphecarpaea bracteata* root nodules (*Devosia sp* - SL43), have been utilized to improve the growth and stress resistance of two major oil seed crops; canola and soybean, under optimal and salt stress conditions. Our findings suggested significant improvements in crop growth of canola and soybean following the application of both biostimulants. Under optimal growth conditions, soybean growth was significantly affected by foliar spray of flavonoids with increases in shoot fresh and dry weight, and leaf area, by 91, 99.5, and 73%, respectively. However, soybean growth was unaffected by flavonoids under salt stress. In contrast, CFS with a meaningful capacity to mitigate the negative effects of salinity stress improved soybean shoot fresh biomass, dry biomass, and leaf area by 128, 163 and 194%, respectively, under salt stress conditions. Canola was less responsive to both biostimulants, except for canola root variables which were substantially improved by flavonoid spray. Since this was the first assessment of these materials as foliar sprays, we strongly encourage further experimentation to confirm the findings reported here and to determine the full range of applicability of each of these potential technologies.

## 1 Introduction

Salinity is an abiotic environmental stress that substantially hinders plant growth, and can have devastating effects on global crop productivity (Türkan and Demiral, 2009; Hasanuzzaman et al., 2013; Naamala and Smith, 2021). Improper irrigation and poor cultivation practices together with extreme environmental fluctuations are the main contributors to excess salt depositions leading to root-zone salinization. The conditions are predominantly critical in arid and semi-arid regions where dry climate progressively promotes translocation and deposition of salts in the upper levels of the soil profile. By the mid-21^st^ century, it is estimated that as much as 50% of the arable land could be lost, no longer suitable for cropping due to salt depositions beyond the critical level (Mahajan and Tuteja, 2005; Islam et al., 2019). As of now, a total of 1125 million ha of agricultural land have been reported to be severely affected by salinity (Islam et al., 2019); as climate change conditions continue to develop they are also likely to contribute to soil salinization (Corwin, 2021). In order to address this potential problem, sustainable and environmentally friendly techniques must be incorporated into our production systems, to meet the dual challenges of crop productivity and environmental stability. Therefore, bio-based or nature-based techniques should be given the utmost importance and preference, especially at this crucial time of increasing climate change conditions.

Flavonoids have been found to improve plant growth through functions within plant tissues and outside, by attracting or repelling beneficial or harmful entities (Tsai and Phillips, 1991; Mandal et al., 2010). Much of the research regarding flavonoid use has been conducted to provoke plant defense systems under unfavorable biotic and abiotic conditions (Nakabayashi et al., 2014; Davies et al., 2018). The increased accumulation of flavonoids in specialized cells or sites (cell wall and membranes) is a plant metabolic strategy to counter the oxidative damage incurred as a secondary effect of such abiotic stresses (Di Ferdinando et al., 2012). Although, there are very few published examples of exogenous application of flavonoids, their increased production and accumulation in plants, as triggered by environmental stresses, have been studied extensively and reported as a plant-adapted strategy to combat the adverse and/or secondary effects of abiotic stresses (Eryılmaz, 2006; Xu et al., 2020). However, the accumulation and biosynthesis of such stress mediators in plants may be reduced when a biostimulant related to stress tolerance capability is applied exogenously (Loubser and Hills, 2020). This could be an energy-saving strategy for plants, as biosynthesis of such compounds requires substantial energy input, whereas utilizing similar compounds received externally, is more feasible and requires comparably lower energy utilization.

In addition to phytochemicals, microbe-based compounds have a key place in agricultural sustainability. As of now, we have just begun to understand the utilization of microbe-based compounds and their importance in plant stress amelioration, however, knowledge in this area is growing, and more interest is developing among those concerned with sustainability. This technology has possible advantages over the direct use of microbial cells, given that they are easy to store and handle, required in minute concentrations, and could bypass limitations that are associated with the use of inoculants (Naamala and Smith, 2021). Microbial compounds have been reported to have growth-stimulating effects even under conditions with extreme environmental limitations (Fincheira et al., 2021). Thuricin 17 and lipo-chitooligosaccharide (LCO - microbe-to-plant signal compounds) are the most extensively studied microbial compounds for their beneficial effects on plant growth and resistance (Nazari and Smith, 2020). For example, the application of thuricin 17 significantly improved crop growth by increasing above-ground and below-ground fresh and dry biomass of corn and soybean (Lee et al., 2009); induced soybean resistance by lignification-related and antioxidant enzyme production (Jung et al., 2011); moderated salt stress in *A. thaliana* by activating antioxidant metabolism pathways including increased levels of IAA and SA (Subramanian, 2014; Subramanian et al., 2016b); promoted root growth and dry biomass of canola under low temperature and salt stresses (Schwinghamer et al., 2016); and improved root growth and water uptake in soybean under drought conditions (Prudent et al., 2015). Likewise, seed treatment and foliar spray with LCO (nod factors) in greenhouse and field trials improved root and shoot growth, and yield of soybean, corn, and cotton (Smith et al., 2011). Studies on exogenous application of LCOs demonstrated induced resistance to salt stress in an array of plant species including *Arabidopsis*, soybean (Subramanian et al., 2016a), blackgram (Nandhini et al., 2018), and maize (Nandhini et al., 2016; Nandhini and Somasundaram, 2018). Interestingly, in addition to these being environmentally friendly technologies, they are reasonably cost and time effective as the reported signal compounds are generall found to perform at their best when applied at very low concentrations (Naamala and Smith, 2021).

The application of bio-based compounds has potential to sustainably improve crop production, however, their incorporation into agricultural systems has been very limited. Although their application is gradually increasing, and growers are developing more interest in sustainability, there is still a gap to be filled. In this study, two biostimulants: CFS (SL43) and flavonoids; after successful trials as seed treatments (Shah et al., 2022), were evaluated for their growth stimulatory effects as foliar sprays. The purpose of this study was to determine whether or not these products can be used on crop foliage, and how effective they are in improving the growth and resistance of canola and soybean under both optimal and salt stress growth conditions.

## 2 Materials and methods

### 2.1 Experimental setup and growing conditions

The study was conducted as pot experiments in a greenhouse at 25 ± 3 °C, illuminated at 800-1000 µmoles.m^-2^.sec^-1^ in a 16:8 h day-night cycle. One-time salt stress was established by adding 300 mL of salt solution to the potting medium at the time of seeding. Plants were watered twice a week or every 2^nd^ day during warmer and sunny days, and fertilized every week with full Hoagland solution. The experimental layout followed a completely randomized design with 4 replications of each treatment, and the experiment was repeated twice.

#### 2.1.1 Foliar application of flavonoids

For the foliar application of flavonoids, a flavonoid solution was made, containing 20% m/v flavonoids (extracted from citrus fruits; Brand: Axenic, ordered from https://www.amazon.ca/gp/product/B07M9X4QJQ/ref=ppx_yo_dt_b_asin_title_o03_s00?ie=UTF8&psc=1). For canola, three seeds were planted at a 3 cm depth in a 25 cm pot filled with Agromix – Teris, Quebec (planting material) followed by irrigation with 300 mL water for treatments under optimal growth conditions, and 300 mL of a salt solution containing 150 mM NaCl for salt-stressed growth conditions. Six to eight days after emergence (at the first true leaf stage), each pot was thinned to one plant, retaining the most uniform plants across all the pots. The canola seedlings were sprayed at the 4-5 leaf stage with one of three levels of flavonoids: 100, 200 and 300 mL ha^-1^ (**Table 1**). In order to reduce the surface tension of the spray solution, and to increase its adhesive attachment with the waxy layer of the leaf surface, Tween^®^ 20 (Fisher’s bioreagents), a surfactant, was added to the solution at 1 mL L^-1^. The same general experimental protocols were followed for soybean, with the following exceptions. After 5-8 days, soybean seedlings were thinned to one per pot at the VC (unifoliate leaves unfolded so leaf edges are not touching) growth stage. Since soybean is more sensitive to salt stress than canola, the level of salt stress was reduced to 120 mM NaCl, which was established by adding 300 mL of salt solution to pots (at first irrigation) allocated to salt stress treatments. In a preliminary experiment for phytotoxicity assessment, soybean plants showed toxic symptoms when sprayed with the same concentration of flavonoids as canola. Therefore, reduced doses of flavonoids were selected for foliar spray on soybean at 50, 100 and 150 mL ha^-1^, and spray-applied at 3-4 trifoliate stage.

**Table 1:** Treatments – Foliar application of flavonoids on canola and soybean.

Treatments (Foliar spray of flavonoids)	
Canola	Soybean
Control (optimal growth) Control (150 mM NaCl) Fl-1 (100 mL ha^-1^) Fl-2 (200 mL ha^-1^) Fl-3 (300 mL ha^-1^) Fl-1 (100 mL ha^-1^) + 150 mM NaCl Fl-2 (200 mL ha^-1^) + 150 mM NaCl Fl-3 (300 mL ha^-1^) + 150 mM NaCl	Control (optimal growth) Control (120 mM NaCl) Fl-1 (50 mL ha^-1^) Fl-2 (100 mL ha^-1^) Fl-3 (150 mL ha^-1^) Fl-1 (50 mL ha^-1^) + 120 mM NaCl Fl-2 (100 mL ha^-1^) + 120 mM NaCl Fl-3 (150 mL ha^-1^) + 120 mM NaCl

#### 2.1.2 Foliar solutions of flavonoids

The doses for flavonoid foliar spray are given on a per ha basis. Since the experiment was conducted in the pots (foliar spraying was conducted using a hand atomizer with a maximum capacity of 50 mL), the doses have been converted to match the desired per ha dose using the conversion formula below. For field applications, flavonoid solutions should be made by mixing the flavonoid concentrate (treatment) with 100 L of water.

Taking treatment “100 mL ha^-1^” as an example:


$$\frac{100\;mL\;flavonoid\;concentrate\;or\;dose\;}{x\;\left(required\;flavonoid\;conc.\;mixed\;to\;50mL\right)}=\frac{100\;L\;\left(solution\;for\;one\;hectare\right)}{50\;mL\;\left(max\;capacity\;of\;hand\;automizer\right)}$$

x=0.05 mL

=> 0.05 *mL* mixed with 50 *mL* water will give the same flavonoid concentrate as 100 mL mixed with 100 L water; sprayed on a 1-ha crop canopy. However, under field conditions, the overall volume is subject to change depending on the crop type, growth stage and crop cover. In our case, we have estimated that each plant received 1 – 2 mL of biostimulant solution on foliage.

#### 2.1.3 Foliar application of CFS

For the foliar application of cell-free supernatant, a bacterial extract material was prepared in from cultures of *Devosia* sp. strain (SL43), isolated from *Amphicarpaea bracteate* plants growing wild along the shore of Lac St. Louis, Sainte-Anne-de-Bellevue, Quebec, Canada. Three dilutions were prepared from the extract material, and selected for foliar spray doses on canola and soybean based on our seed germination experiments with the extracted material (Shah et al., 2022); minimum (1:100), intermediate (1:500) and maximum (1:1000) dilutions. (**Table 2**). CFS was sprayed on canola and soybean at the 4-5 leaf stage and 3-4 trifoliate stage, respectively. The salt stress levels, growth conditions and experimental design for both crops were the same as used in the previous experiment.

**Table 2:** Treatments – Foliar application of CFS on canola and soybean.

Treatments (Foliar spray of CFS)	
Canola	Soybean
Control Control (150 mM NaCl) CFS-1 (1:100) CFS-2 (1:500) CFS-3 (1:1000) CFS -1 (1:100) + 150 mM NaCl CFS -2 (1:500) + 150 mM NaCl CFS -3 (1:1000) + 150 mM NaCl	Control Control (120 mM NaCl) CFS -1 (1:100) CFS -2 (1:500) CFS -3 (1:1000) CFS -1 (1:100) + 120 mM NaCl CFS -2 (1:500) + 120 mM NaCl CFS -3 (1:1000) + 120 mM NaCl

### 2.2 Sampling and data collection

A one-time sampling was carried out at the vegetative growth stages of both canola and soybean. For canola, the harvest was carried out at the 8-10 leaf stage, and for soybean, harvesting was done at V5-V6 growth stages (10 days after spray application in both cases). Plant growth variables including shoot fresh weight (g), shoot dry weight (g), leaf area (cm^2^), root fresh weight (g), root dry weight (g), root length (g) and root volume (cm^3^) were measured. The fresh weight of above-ground biomass (g) was analyzed immediately after plant harvest, followed by leaf area (cm^2^) measurement using an LI-3100C leaf area meter (LI-COR, USA). The shoot samples were later placed into a drying oven at 65 ^0^C for two days for the determination of shoot dry biomass (g). Below-ground fresh biomass was recorded after washing the root samples followed by drying with paper towels. After that, roots were scanned (EPSON Expression 11000XL) and analyzed using the WinRHIZO™ (Regent Instruments Inc.) image analysis platform for root measurements.

### 2.3 Data analysis

All experiments were structured following a completely randomized design. The experimental data sets (each comprising of 4 technical replicates) were pooled for data analysis using SAS 9.4 (SAS Institute Inc., Cary, NC, USA). The data was analyzed by one-way ANOVA using Proc GLM and Tukey’s multiple means comparison to determine statistical differences between treatments, at the 95% confidence level. Data that were significant at *p*< 0.05 were considered for response description. When the data were numerically higher or lower as compared to control with no statistical significance, percentage differences were calculated to support the treatment responses, since plants’ responses to biostimulants are very subtle but still are biologically significant.

## 3 Results

### 3.1 Responses of soybean growth variables to flavonoid and CFS spray applications

#### 3.1.1 Soybean above-ground biomass

**Figure 1:**
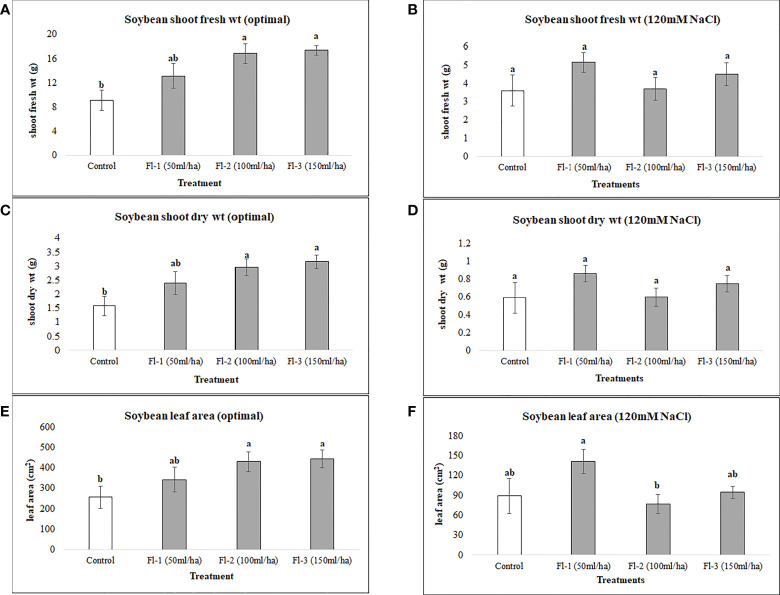
Soybean above ground biomass affected by flavonoids **(A)** shoot fresh wt - optimal **(B)** shoot fresh wt – 120 mM NaCl **(C)** shoot dry wt - optimal **(D)** shoot dry wt – 120 mM NaCl **(E)** leaf area - optimal **(F)** leaf area – 120 mM NaCl. The data represents the mean values of 8 replications (n=8) ± standard error. Different letters indicate values determined by Tukey’s multiple mean comparison to be significantly different (p< 0.05) from each other.

**Figure 2:**
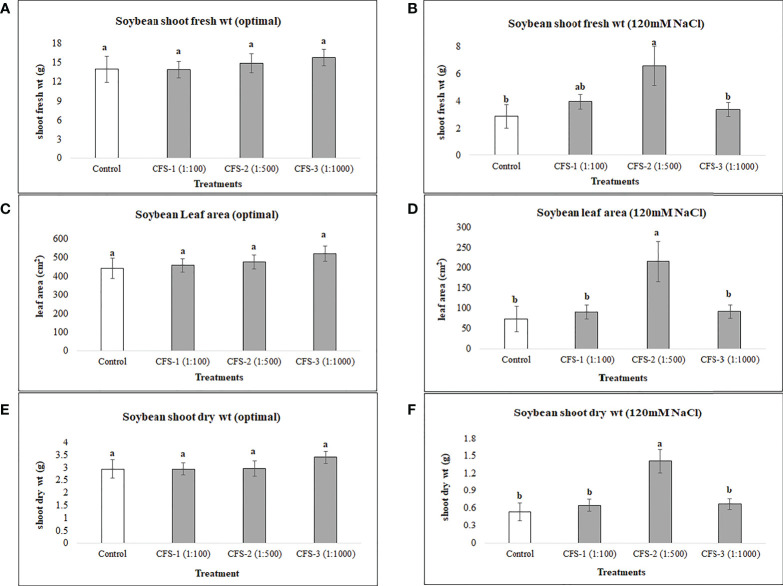
Soybean above ground biomass affected by CFS **(A)** shoot fresh wt - optimal **(B)** shoot fresh wt – 120 mM NaCl **(C)** shoot dry wt - optimal **(D)** shoot dry wt – 120 mM NaCl **(E)** leaf area - optimal **(F)** leaf area – 120 mM NaCl. The data represents the mean values of 8 replications (n=8) ± standard error. Different letters indicate values determined by Tukey’s multiple mean comparison to be significantly different (p< 0.05) from each other.

Under optimal conditions, soybean growth was significantly improved by foliar spray of flavonoids (*P* = 0.0030); however, no significant differences could be detected under salt stress (120 mM NaCl). Under optimal growth conditions, soybean shoot fresh weight, leaf area and shoot dry weight were significantly increased by 91, 73 and 99%, respectively, over the controls. Treatment Fl-3 (150 mL ha^-1^) resulted in the greatest increases, followed by Fl-2 (100 mL ha^-1^) and Fl-1 (50 mL ha^-1^), illustrating that soybean is likely more responsive to higher flavonoid concentrations under optimal growth conditions (**Figure 1**). In contrast to flavonoid application, CFS with greater salinity stress mitigation effects, significantly improved soybean above-ground fresh and dry biomass over controls by 127 and 163% under salt stress (P = 0.038). In addition, a nearly two-fold (194%) increase in soybean leaf area was detected when compared with the control. No significant differences occurred for CFS treatments under optimal growth conditions (**Figure 2**).

#### 3.1.2 Soybean below-ground biomass

**Figure 3:**
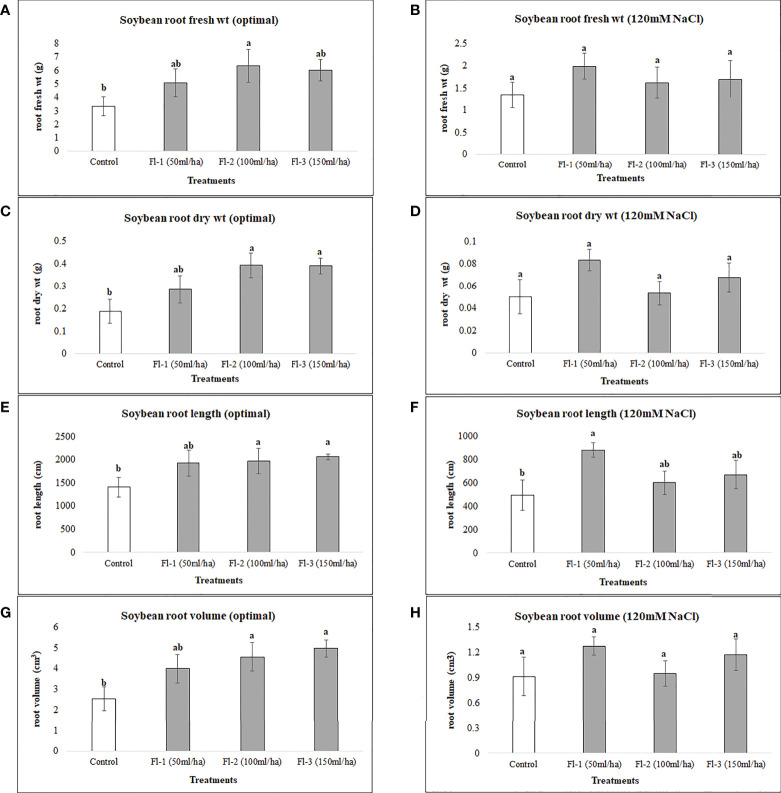
Soybean root variables affected by flavonoids **(A)** root fresh wt - optimal **(B)** root fresh wt – 120 mM NaCl **(C)** root dry wt - optimal **(D)** root dry wt – 120 mM NaCl **(E)** root length - optimal **(F)** root length – 120 mM NaCl **(G)** root volume - optimal **(H)** root volume – 120 mM NaCl. The data represents the mean values of 8 replications (n=8) ± standard error. Different letters indicate values determined by Tukey’s multiple mean comparison to be significantly different (p< 0.05) from each other .

Soybean root variables were more responsive to flavonoid treatments under optimal growth conditions as soybean root fresh and dry root biomass were increased by 90 and 109%, respectively, over the controls. In contrast, no significant differences could be seen for these variables under salt stress for flavonoid treatments. Soybean root length for biostimulant treatments, however, was surprisingly high under both optimal and salt stress conditions when compared with controls. Under optimal growth conditions, increases in root length resulted from treatment with Fl-2 and Fl-3, by 46.5 and 40.3%, respectively. Under salt stress, root length was increased by 77.6% by treatment with Fl-1 with an average root length of 881.4 cm, as compared to the control with 496.4 cm (**Figure 3**).

**Figure 4:**
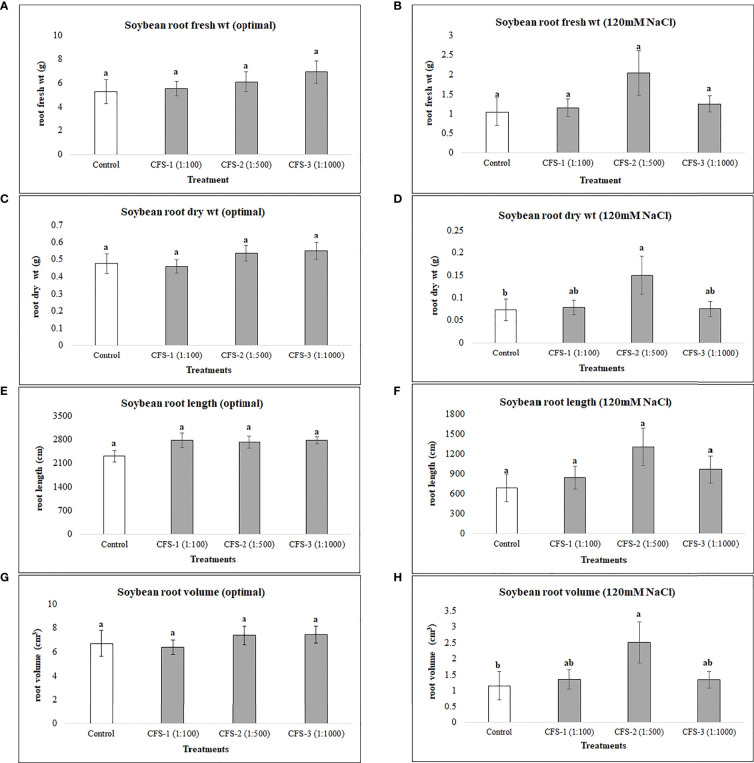
Soybean root variables affected by CFS **(A)** root fresh wt - optimal **(B)** root fresh wt – 120 mM NaCl **(C)** root dry wt - optimal **(D)** root dry wt – 120 mM NaCl **(E)** root length - optimal **(F)** root length – 120 mM NaCl **(G)** root volume - optimal **(H)** root volume – 120 mM NaCl. The data represents the mean values of 8 replications (n=8) ± standard error. Different letters indicate values determined by Tukey’s multiple mean comparison to be significantly different (p< 0.05) from each other.

On the other hand, CFS was found statistically insignificant, with no or minimal effect on soybean root variables under optimal growth conditions. However, under salt stress, root dry biomass and root volume were substantially improved, by 150 and 117%, respectively, when compared with the control treatment (**Figure 4**)

### 3.2 Responses of Canola growth variables to flavonoids and CFS spray

#### 3.2.1 Canola above-ground biomass

**Figure 5:**
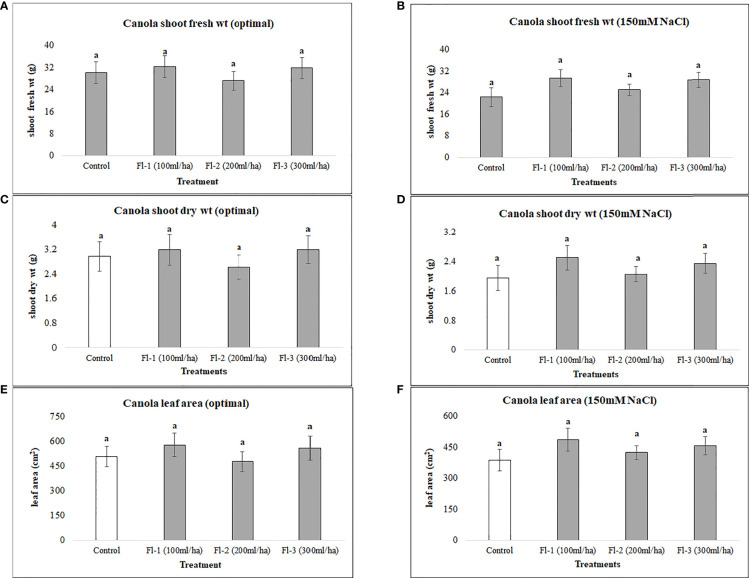
Canola above ground biomass affected by flavonoids **(A)** shoot fresh wt - optimal **(B)** shoot fresh wt – 150 mM NaCl **(C)** shoot dry wt - optimal **(D)** shoot dry wt – 150 mM NaCl **(E)** leaf area - optimal **(F)** leaf area – 150 mM. The data represents the mean values of 8 replications (n=8) ± standard error. Different letters indicate values determined by Tukey’s multiple mean comparison to be significantly different (p< 0.05) from each other.

**Figure 6:**
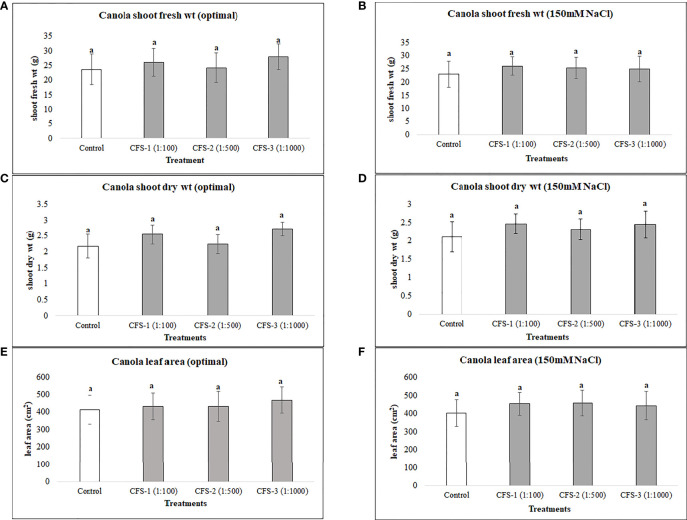
Canola above ground biomass affected by CFS **(A)** shoot fresh wt - optimal **(B)** shoot fresh wt – 150 mM NaCl **(C)** shoot dry wt - optimal **(D)** shoot dry wt – 150 mM NaCl **(E)** leaf area - optimal **(F)** leaf area – 150 mM NaCl. The data represents the mean values of 8 replications (n=8) ± standard error. Different letters indicate values determined by Tukey’s multiple mean comparison to be significantly different (p< 0.05) from each other.

Canola was generally less responsive to both flavonoids and CFS application than soybean, as none of the flavonoid or CFS treatments resulted in significant effects on canola above-ground fresh and dry biomass under both salt stress (150 mM NaCl) and optimal growth conditions. Under optimal growth conditions, the average biomass of canola for flavonoid treatments was very similar to that of the control, with around only a 6% increase in canola fresh and dry biomass. Nevertheless, flavonoids showed some stress amelioration effects; although not statistically significant, but caused increases in canola shoot fresh and dry biomass under salt stress, by 25 and 28%, respectively, as compared to the control (**Figure 5**). CFS on the other hand, with less stimulatory effects than that of flavonoids, showed minimal or no effect on canola above-ground biomass under both optimal and salt stress conditions. However, comparing the growth stimulations for both biostimulants, canola seems to be more responsive to flavonoids than CFS dilutions (**Figure 6**).

#### 3.2.2 Canola below-ground biomass

**Figure 7:**
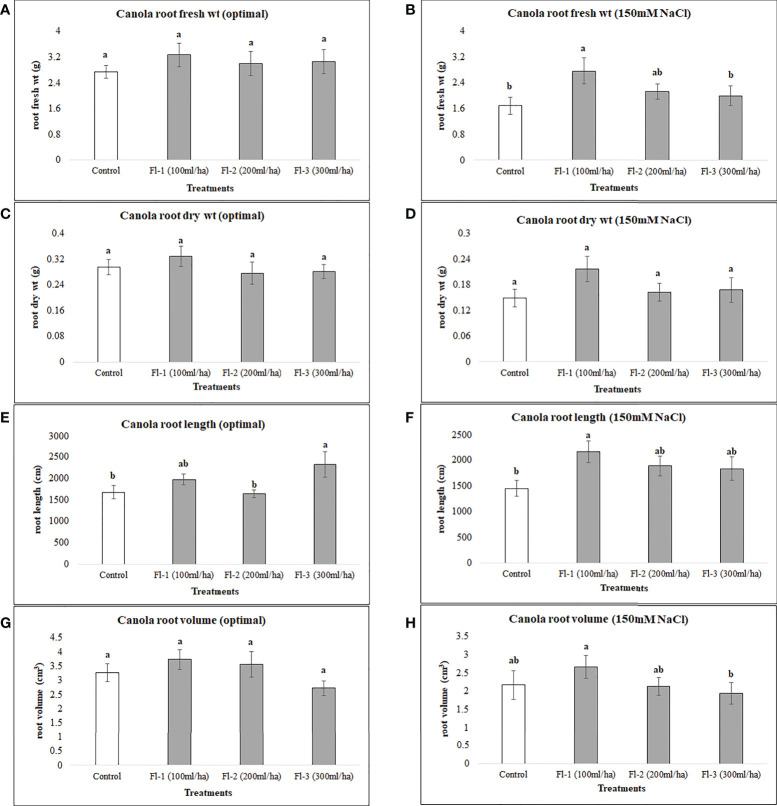
Canola root variables affected by flavonoids **(A)** root fresh wt - optimal **(B)** root fresh wt – 150 mM NaCl **(C)** root dry wt - optimal **(D)** root dry wt – 150 mM NaCl **(E)** root length - optimal **(F)** root length – 150 mM NaCl **(G)** root volume - optimal **(H)** root volume – 150 mM NaCl. The data represents the mean values of 8 replications (n=8) ± standard error. Different letters indicate values determined by Tukey’s multiple mean comparison to be significantly different (p< 0.05) from each other.

The below-ground biomass of canola under optimal growth conditions was unaffected by the foliar application of flavonoids, as no significant differences under optimal growth conditions were detected for flavonoid treatments, except for canola root length which was significantly higher than the control, by 38%. The growth responses of the two crops to flavonoid foliar spray were generally quite different, however, increases in root length of both crops following the application of flavonoids indicates a strong link between flavonoids and crop root growth, particularly root length. Under salt stress, flavonoids caused much greater increases in root fresh weight, root length and volume, increasing them by 63.7, 49 and 117%, respectively, over the control (**Figure 7**). In contrast, CFS application did not affect canola below-ground growth under either optimal or salt stress conditions.

## 4 Discussion

### 4.1 Foliar spray of flavonoids

Flavonoids are strong antioxidants and have strong stress amelioration effects against an array of biological and environmental stresses (Chutipaijit et al., 2009; Koskimäki et al., 2009; Fini et al., 2011; Khlestkina, 2013; Shah and Smith, 2020). However, the exogenous application of flavonoids has rarely been investigated. Therefore, this study was conducted to determine the growth-stimulating effects of citrus-extracted flavonoids on canola and soybean under salt stress and optimal growth conditions.

In the case of soybean, significant improvements in growth variables occurred under optimal growth conditions, following the foliar application of flavonoids. For example, soybean shoot fresh weight, dry weight, and leaf area were significantly increased, by 91, 99.5 and 73%, respectively, when compared with control plants. In addition, root variables, for instance, root fresh and dry biomass, were improved by 90 and 109%, respectively, over the control. In addition, there were reasonably consistent increases in soybean growth observed following flavonoid treatments under salt stress; for instance, soybean shoot fresh weight, dry weight and leaf area were increased under salt stress (120 mM NaCl) by 43, 46 and 57%, respectively, over the control. However, these differences were not statistically different from the control, which could be due to the small sample size and high treatment-response variability. An experiment with a larger sample size may reduce the data variation within the treatments.

In addition, under optimal growth conditions, a consistent increase in growth variables resulted from flavonoid treatment at greater concentrations. More substantial results were achieved by the treatment containing the highest flavonoid concentration; Fl – 3 (150 mL ha^-1^), followed by Fl – 2 (100 mL ha^-1^) and Fl – 1 (50 mL ha^-1^). In contrast, the greatest responses under salt stress were for Fl-1 (50 mL ha^-1^) indicating that soybean responded better to high flavonoid treatment under optimal growth conditions, however, lower concentrations provoked responses under salt stress. Flavonoid treatment did not increase canola growth under both optimal growth conditions and salt stress (150 mM NaCl), except for canola root variables (root fresh weight and root length) which were significantly enhanced over the control treatment. Interestingly, canola responses to flavonoid treatments were similar to those of soybean, in that the lower doses of flavonoid (100 mL ha^-1^) provided the best effects under salt stress. The same pattern was also observed under optimal conditions, indicating that canola responded better to the lowest doses of flavonoids, irrespective of growth conditions (optimal or salt stress). It is possible that the optimal dose for flavonoids, in these conditions, is lower than those evaluated here. Therefore, further experiments are advised to be conducted using lower flavonoid doses to confirm this supposition.

It seems that, like flavonoids under salt stress, more effects occur for below-ground biomass, as similar responses occurred for both the crops under salt stress. Overall, in this research, we have seen a range of unexpected, and complex responses from both crops following flavonoid treatments, which has complicated response outcomes. This suggests that these treatment responses are both dose and crop-type dependent. Conclusively, our findings support the suggestion of Dieter Treutter (2006), that “the multi-functionality of these compounds (flavonoids), however, often complicates the interpretation of experimental results but, finally, it supports their importance” (Treutter, 2006).

### 4.2 Foliar spray of CFS

Foliar spray of microbe-based products is a recent approach, and is not commonly practiced. The foliar spray of thuricin 17; a bacteriocin produced by *B. thuringiensis*, increased the leaf area, photosynthetic rate and plant dry weight of soybean and corn (Lee et al., 2009). In addition, increased potential has been observed for this bacteriocin and a bacterium-produced nod factor (LCO) in promoting soybean growth under stressful conditions (Gautam et al., 2016). In this part of the research, the CFS, after successful trials on seed germination of soybean and canola (Shah et al., 2022), was applied as foliar spray with the thinking that it would increase the growth of both crops under salt stress and optimal growth conditions. Arguably, soybean growth was unaffected by CFS under optimal growth conditions, however, this is not overly surprising as many of the bacteria-to-plant signal compounds show little or no effects in the absence of stress, and large effects in the presence of stress (Subramanian et al., 2016b), particularly at lower concentrations (Gautam et al., 2016). This experiment revealed interesting and unexpected results. Unlike flavonoids, CFS had greater effects under salinity stress conditions, as under salt stress, soybean growth was significantly improved by CFS spray, indicating that it may contain active materials that induce resistance against salt stress, and possibly other abiotic stresses. The mode of action for this effect is unknown; based on current understandings we can speculate, but we cannot be certain about this until further experimentation including molecular or “omics” studies has been conducted, to provide deeper insight and understanding regarding how these compounds function within plant tissues including through modulating plants defense systems.

Canola growth, in contrast to soybean, showed no significant responses to CFS application under both salt stress (150 mM NaCl) and optimal growth conditions. This could be because the CFS was extracted from a novel strain of a nodule-associated bacteria (*Devosia* sp SL43*)* isolated from a native legume (*Amphicarpaea bracteata*); from an evolutionary perspective, it might be the case that it is less or not effective on non-legumes. However, more crop plants should be evaluated before a conclusion is reached on this. As this is the very first time we have experimented with this material, there is still a considerable amount of research left to be conducted.

## 5 Conclusions

Foliar spray of flavonoids substantially enhanced soybean growth under optimal growth conditions, however, under salinity stress, no differences were detected related to flavonoid treatments. In contrast, CFS application under salinity stress significantly improved soybean growth, but caused no significant improvements under optimal growth conditions. Canola however showed no growth responses to flavonoids or CFS, except for the root variables that were enhanced by flavonoid treatments under salt stress. The differences in crop responses to flavonoids and CFS under optimal conditions suggests effects of the strong evolutionary relationship of soybean to flavonoids, especially in the context of root nodulation. This component makes soybean respond to flavonoids positively to the below-ground microbes. Similarly, the CFS derived from a legume endophyte alleviated salt stress in soybean suggesting that the plant growth stimulating responses in soybean might have some elements of coevolution for abiotic stress that we do not yet understand. From the microbial perspective, the bacterium was isolated from Amphecarpaea bracteate that grows well in moist soils, potentially under anaerobic conditions, making both the plant and the associated bacteria, a repository for microbes evolved under abiotic stress, of which, Devosia is reported to be a good biostimulant in previous studies from our laboratory. Further experiments to understand the molecular aspects of these responses, both from the plant and the bacterial perspectives, could provide further insights into the mode of action of these biostimulants.

## Data availability statement

The raw data supporting the conclusions of this article will be made available by the authors, without undue reservation.

## Author contributions

This research was conducted and written by AS. Statistical data analysis was carried out by SSas her contribution to this manuscript. In addition, she prepared the tables and helped in editing the manuscript. The manuscript was edited and proofread by DS, who also contributed to the design of the overall project and provided intellectual context and financial support. All authors contributed to the article and approved the submitted version.

## Funding

The authors would like to acknowledge the support for this manuscript was provided through the Biomass Canada Cluster (BMC), which is funded through Agriculture and Agri-Food Canada’s AgriScience program and industry partners.

## Conflict of interest

The authors declare that the research was conducted in the absence of any commercial or financial relationships that could be construed as a potential conflict of interest.

## Publisher’s note

All claims expressed in this article are solely those of the authors and do not necessarily represent those of their affiliated organizations, or those of the publisher, the editors and the reviewers. Any product that may be evaluated in this article, or claim that may be made by its manufacturer, is not guaranteed or endorsed by the publisher.
