# Supplementary material for: Flavonoids and Devosia sp SL43 cell-free supernatant increase early plant growth under salt stress and optimal growth conditions
Source: Front Plant Sci. 2022 Nov 10;13:1030985. doi: 10.3389/fpls.2022.1030985 (PMC9690568; doi:10.3389/fpls.2022.1030985)
Supplement: Supplementary file 1 [file DataSheet_1.pdf]

Supplementary Tables

Table 1: Soybean growth responses to the foliar spray of flavonoids under salt stress (120 mM NaCl) and optimal growth condition

Soybean growth variables (foliar spray of flavonoids) – optimal conditions

**Treatments**	Shoot FW (g) ± SE	Shoot DW (g) ± SE	Leaf area (cm^2) ± SE	Root FW (g) ± SE	Root DW (g) ± SE	Root length (cm) ± SE	Root volume (cm^3) ± SE
***p*-value**	.0030	.0090	.0575	.1435	.0256	.1821	.0381
Ctrl	$9.09^{b} \pm 1.70$	$1.59^{b} \pm 0.35$	$255.60^{b} \pm 54.73$	$3.35^{b} \pm 0.70$	$0.188^{b} \pm 0.052$	$1407.25^{b} \pm 213.82$	$2.523^{b} \pm 0.581$
Fl 50 mL ha^{-1}	$13.15^{ab} \pm 1.99$	$2.40^{ab} \pm 0.40$	$342.00^{ab} \pm 59.95$	$5.09^{ab} \pm 1.40$	$0.285^{ab} \pm 0.060$	$1929.50^{ab} \pm 280.17$	$3.992^{ab} \pm 0.677$
Fl 100 mL ha^{-1}	$16.83^{a} \pm 1.60$	$2.96^{a} \pm 0.30$	$429.43^{a} \pm 48.32$	$6.35^{a} \pm 1.21$	$0.393^{a} \pm 0.053$	$1975.25^{a} \pm 272.08$	$4.569^{a} \pm 0.689$
Fl 150 mL ha^{-1}	$17.35^{a} \pm 0.75$	$3.17^{a} \pm 0.24$	$443.77^{a} \pm 43.54$	$6.02^{ab} \pm 0.81$	$0.390^{a} \pm 0.035$	$2062.25^{a} \pm 59.11$	$4.969^{a} \pm 0.399$

Soybean growth variables (foliar spray of flavonoids) – 120 mM NaCl condition

**Treatments**	Shoot FW (g) ± SE	Shoot DW (g) ± SE	Leaf area (cm^2) ± SE	Root FW (g) ± SE	Root DW (g) ± SE	Root length (cm) ± SE	Root volume (cm^3) ± SE
***p*-value**	.3292	.3346	.0895	.6209	.2421	.0968	.4101
Ctrl	$3.60^{a} \pm 0.85$	$0.59^{a} \pm 0.17$	$89.25^{ab} \pm 26.27$	$1.34^{a} \pm 0.29$	$0.050^{a} \pm 0.015$	$496.38^{b} \pm 129.42$	$0.914^{a} \pm 0.229$
Fl 50 mL ha^{-1}	$5.15^{a} \pm 0.53$	$0.86^{a} \pm 0.09$	$140.92^{a} \pm 18.26$	$1.99^{a} \pm 0.29$	$0.083^{a} \pm 0.010$	$881.38^{a} \pm 62.81$	$1.275^{a} \pm 0.107$
Fl 100 mL ha^{-1}	$3.70^{a} \pm 0.62$	$0.60^{a} \pm 0.11$	$76.98^{b} \pm 14.21$	$1.62^{a} \pm 0.36$	$0.054^{a} \pm 0.011$	$600.63^{ab} \pm 101.14$	$0.950^{a} \pm 0.148$
Fl 150 mL ha^{-1}	$4.50^{a} \pm 0.62$	$0.75^{a} \pm 0.09$	$94.81^{ab} \pm 9.01$	$1.69^{a} \pm 0.41$	$0.068^{a} \pm 0.013$	$670.38^{ab} \pm 121.21$	$1.173^{a} \pm 0.189$

Table 2: Soybean growth responses to the foliar spray of **CFS** under salt stress (120mM NaCl) and optimal growth condition

Soybean growth variables (foliar spray of CFS) – optimal conditions

**Treatments**	Shoot FW (g) ± SE	Shoot DW (g) ± SE	Leaf area (cm^2) ± SE	Root FW (g) ± SE	Root DW (g) ± SE	Root length (cm) ± SE	Root volume (cm^3) ± SE
***p*-value**	0.7968	0.9998	0.6069	0.5506	0.4781	0.1657	0.7633
Ctrl	13.96[a] ± 1.99	2.94[a] ± 0.37	444.04[a] ± 54.67	5.27[a] ± 1.00	0.47[a] ± 0.06	2312.88[a] ± 166.94	6.71[a] ± 1.09
Fl 50 mL ha^{-1}	13.95[a] ± 1.30	2.94[a] ± 0.24	458.41[a] ± 35.76	5.53[a] ± 0.62	0.46[a] ± 0.04	2784.50[a] ± 215.08	6.40[a] ± 0.59
Fl 100 mL ha^{-1}	14.90[a] ± 1.45	2.96[a] ± 0.31	477.37[a] ± 38.40	6.11[a] ± 0.85	0.54[a] ± 0.05	2730.25[a] ± 172.40	7.38[a] ± 0.80
Fl 150 mL ha^{-1}	15.83[a] ± 1.30	2.98[a] ± 0.48	521.56[a] ± 39.58	6.92[a] ± 0.93	0.55[a] ± 0.05	2783.00[a] ± 100.30	7.44[a] ± 0.72

Soybean growth variables (foliar spray of CFS) – 120 mM NaCl condition

**Treatments**	Shoot FW (g) ± SE	Shoot DW (g) ± SE	Leaf area (cm^2) ± SE	Root FW (g) ± SE	Root DW (g) ± SE	Root length (cm) ± SE	Root volume (cm^3) ± SE
***p*-value**	0.0379	0.0007	0.0132	0.2386	0.1160	0.2547	0.1345
Ctrl	2.89[b] ± 0.86	0.54[b] ± 0.15	73.24[b] ± 31.81	1.05[a] ± 0.36	0.06[b] ± 0.02	689.25[a] ± 210.07	1.16[b] ± 0.44
Fl 50 mL ha^{-1}	3.95[ab] ± 0.55	0.65[b] ± 0.10	90.53[b] ± 17.77	1.16[a] ± 0.22	0.08[ab] ± 0.02	848.75[a] ± 172.97	1.36[ab] ± 0.30
Fl 100 mL ha^{-1}	6.58[a] ± 1.43	1.41[a] ± 0.21	215.38[a] ± 49.24	2.04[a] ± 0.57	0.15[a] ± 0.04	1309.50[a] ± 277.79	2.52[a] ± 0.64
Fl 150 mL ha^{-1}	3.38[b] ± 0.52	0.67[b] ± 0.09	91.82[b] ± 16.68	1.25[a] ± 0.21	0.08[ab] ± 0.02	968.88[a] ± 205.87	1.35[ab] ± 0.26

Table 3: Canola growth responses to the foliar spray of **flavonoids** under salt stress (150 mM NaCl) and optimal growth condition

Canola growth variables (foliar spray of Flavonoids) – optimal conditions

**Treatments**	Shoot FW (g) ± SE	Shoot DW (g) ± SE	Leaf area (cm^2) ± SE	Root FW (g) ± SE	Root DW (g) ± SE	Root length (cm) ± SE	Root volume (cm^3) ± SE
***p*-value**	0.7808	0.7996	0.6978	0.7387	0.5684	0.0548*	0.2196
Ctrl	30.15^a ± 3.87	2.98^a ± 0.48	508.41^a ± 63.15	2.74^a ± 0.20	0.29^a ± 0.02	1677.75^b ± 161.25	3.27^a ± 0.32
Fl 100 mL ha^{-1}	32.24^a ± 3.93	3.19^a ± 0.50	579.55^a ± 70.97	3.27^a ± 0.37	0.33^a ± 0.03	1970.38^{ab} ± 125.84	3.73^a ± 0.36
Fl 200 mL ha^{-1}	27.25^a ± 3.45	2.63^a ± 0.40	477.92^a ± 59.61	3.00^a ± 0.38	0.28^a ± 0.04	1645.75^b ± 86.34	3.57^a ± 0.45
Fl 300 mL ha^{-1}	31.86^a ± 3.84	3.19^a ± 0.45	559.50^a ± 73.55	3.06^a ± 0.38	0.28^a ± 0.02	2325.25^a ± 300.80	2.73^a ± 0.27

Canola growth variables (foliar spray of Flavonoids) – 150mM NaCl condition

**Treatments**	Shoot FW (g) ± SE	Shoot DW (g) ± SE	Leaf area (cm^2) ± SE	Root FW (g) ± SE	Root DW (g) ± SE	Root length (cm) ± SE	Root volume (cm^3) ± SE
***p*-value**	0.6435	0.6341	0.5886	0.1032	0.2294	0.1344	0.1345
Ctrl	23.43^a ± 3.30	1.95^a ± 0.34	387.09^a ± 52.71	1.69^b ± 0.27	0.15^a ± 0.02	1449.63^b ± 152.07	1.16^b ± 0.44
Fl 100 mL ha^{-1}	29.33^a ± 3.18	2.50^a ± 0.34	487.04^a ± 55.51	2.76^a ± 0.40	0.22^a ± 0.03	2162.75^a ± 206.09	1.36^{ab} ± 0.30
Fl 200 mL ha^{-1}	25.10^a ± 2.12	2.05^a ± 0.21	424.23^a ± 34.26	2.12^{ab} ± 0.24	0.16^a ± 0.02	1884.63^{ab} ± 186.39	2.52^a ± 0.64
Fl 300 mL ha^{-1}	25.12^a ± 4.42	2.05^a ± 0.39	401.53^a ± 70.08	1.74^b ± 0.38	0.15^a ± 0.03	1601.50^{ab} ± 311.28	1.35^{ab} ± 0.26

Table 4: Canola growth responses to the foliar spray of **CFS** under salt stress (150 mM NaCl) and optimal growth condition

Canola growth variables (foliar spray of CFS) – optimal conditions

**Treatments**	Shoot FW (g) ± SE	Shoot DW (g) ± SE	Leaf area (cm^2) ± SE	Root FW (g) ± SE	Root DW (g) ± SE	Root length (cm) ± SE	Root volume (cm^3) ± SE
***p*-value**	0.9201	0.5508	0.9675	0.7486	0.5901	0.7025	0.7024
Ctrl	23.68^{a} ± 5.20	2.18^{a} ± 0.38	413.00^{a} ± 82.09	2.23^{a} ± 0.26	0.31^{a} ± 0.05	1918.13^{a} ± 166.14	3.07^{a} ± 0.17
Fl 100 mL ha^{-1}	26.08^{a} ± 4.70	2.55^{a} ± 0.31	432.25^{a} ± 75.19	2.09^{a} ± 0.18	0.34^{a} ± 0.03	1943.83^{a} ± 146.04	3.05^{a} ± 0.21
Fl 200 mL ha^{-1}	24.26^{a} ± 4.99	2.24^{a} ± 0.30	431.74^{a} ± 85.86	2.21^{a} ± 0.23	0.34^{a} ± 0.04	1841.60^{a} ± 98.35	3.08^{a} ± 0.21
Fl 300 mL ha^{-1}	27.98^{a} ± 4.28	2.72^{a} ± 0.21	468.61^{a} ± 74.96	2.43^{a} ± 0.21	0.39^{a} ± 0.04	2101.30^{a} ± 204.16	3.38^{a} ± 0.29

Canola growth variables (foliar spray of CFS) – 150mM NaCl condition

**Treatments**	Shoot FW (g) ± SE	Shoot DW (g) ± SE	Leaf area (cm^2) ± SE	Root FW (g) ± SE	Root DW (g) ± SE	Root length (cm) ± SE	Root volume (cm^3) ± SE
***p*-value**	0.9678	0.8980	0.9491	0.8803	0.6190	0.5760	0.1636
Ctrl	23.08^{a} ± 4.96	2.10^{a} ± 0.41	403.92^{a} ± 73.88	1.98^{a} ± 0.23	0.30^{a} ± 0.04	1782.13^{a} ± 94.33	3.02^{ab} ± 0.25
Fl 100 mL ha^{-1}	26.05^{a} ± 3.49	2.45^{a} ± 0.27	453.96^{a} ± 63.55	2.22^{a} ± 0.25	0.37^{a} ± 0.03	1722.63^{a} ± 141.95	3.43^{ab} ± 0.19
Fl 200 mL ha^{-1}	25.34^{a} ± 4.04	2.31^{a} ± 0.29	457.40^{a} ± 71.27	2.25^{a} ± 0.19	0.35^{a} ± 0.03	2018.00^{a} ± 191.26	3.57^{a} ± 0.28
Fl 300 mL ha^{-1}	25.01^{a} ± 4.82	2.14^{a} ± 0.46	443.97^{a} ± 77.54	2.14^{a} ± 0.35	0.31^{a} ± 0.05	1884.25^{a} ± 184.15	2.79^{b} ± 0.33
